# Leptin induces inflammation-related genes in RINm5F insulinoma cells

**DOI:** 10.1186/1471-2199-8-41

**Published:** 2007-05-23

**Authors:** Paul Hekerman, Julia Zeidler, Stefanie Korfmacher, Simone Bamberg-Lemper, Holger Knobelspies, Lennart Zabeau, Jan Tavernier, Walter Becker

**Affiliations:** 1 Institute of Pharmacology and Toxicology, Medical Faculty of the RWTH Aachen University, Wendlingweg 2, 52074 Aachen, Germany; 2 The Flanders Interuniversity Institute for Biotechnology, Department of Medical Protein Research (VIB9), Ghent University, Faculty of Medicine and Health Sciences, A. Baertsoenkaai 3, 9000 Ghent, Belgium

## Abstract

**Background:**

Leptin acts not only on hypothalamic centers to control food intake but has additional functions in peripheral tissues, *e.g*. inhibition of insulin secretion from pancreatic islets. The leptin receptor (LEPRb) is a class I cytokine receptor that mediates activation of STAT transcription factors. In this study, we characterise the regulation of inflammation-related genes by leptin in insulinoma cells and compare the effect of transcriptional regulation by leptin with that of other cytokines.

**Results:**

We have used RINm5F insulinoma cells as a model system for a peripheral target cell of leptin. Six transcripts encoding inflammation-related proteins were found to be upregulated by activation of LEPRb, namely lipocalin-2, pancreatitis-associated protein, preprotachykinin-1, fibrinogen-β, tissue-type plasminogen activator (tPA) and manganese-dependent superoxide dismutase (MnSOD). Four of these transcripts (fibrinogen-β, lipocalin-2, tPA, MnSOD) were also induced by the proinflammatory cytokine interleukin-1β (IL-1β). Interferon-γ alone had no effect on the leptin-induced transcripts but enhanced the upregulation by IL-1β of lipocalin-2, tPA and MnSOD mRNA levels. Experiments with LEPRb point mutants revealed that the upregulation of the inflammation-related genes depended on the presence of tyrosine-1138 which mediates the activation of the transcription factors STAT1 and STAT3. Reporter gene assays showed that leptin induced the expression of preprotachykinin-1 and lipocalin-2 on the level of promoter regulation. Finally, leptin treatment increased caspase 3-like proteolytic activity in RINm5F cells.

**Conclusion:**

The present data show that leptin induces a cytokine-like transcriptional response in RINm5F cells, consistent with the proposed function of leptin as a modulator of immune and inflammatory responses.

## Background

Leptin is a cytokine which is secreted predominantly by white adipose tissue and exerts its effects on appetite and thermogenesis on centers located in the hypothalamus [1]. In addition to these well defined central actions, it has become clear that leptin also has important peripheral effects, including the regulation of the immune system and the modulation of insulin secretion from the pancreas [2-4]. The requirement of leptin for a normal immune response has been characterised in detail in various animal models of autoimmune diseases and experimentally induced inflammation [5,6]. This proinflammatory effect of leptin is at least partially mediated by its direct action on T-lymphocytes, although macrophages and natural killer (NK) cells also appear to be responsive to leptin [5-7].

Two main signalling pathways have been proposed to mediate the inhibitory effect of leptin on insulin secretion. Firstly, leptin causes the PI-3 kinase-dependent activation of phosphodiesterase 3B (PDE3B) and subsequent reduction of intracellular cAMP levels [8]. Secondly, leptin activates ATP-sensitive K^+ ^channels on β-cells and thereby antagonises depolarisation required for insulin secretion [9]. These are both unusual pathways, considering that the leptin receptor (LEPR) belongs to the class I cytokine receptor family and transmits signals mainly *via *the JAK/STAT (Janus kinase/signal transducer and activator of transcription) pathway [10]. LEPR exists in several splicing variants, the longest one (LEPRb) being the only one capable of activating JAK/STAT signalling [11,12]. The effect of leptin on β-cells clearly depends on the presence of the long splicing variant of the leptin receptor (LEPRb), because leptin does not inhibit insulin secretion from islets isolated from *db*/*db *mice which harbour a splice site mutation that specifically affects expression of LEPRb [13]. Ligand binding to LEPRb results in trans-autophosphorylation and thereby activation of the receptor-associated tyrosine kinase JAK2 and subsequent recruitment and activation of STAT factors to phosphorylated tyrosine residues in the cytoplasmic part of LEPRb [10,14]. In rat islet cells and in rodent insulinoma cell lines (RINm5F, MIN6), leptin has been shown to stimulate tyrosyl phosphorylation of STAT1, STAT3, STAT5 and dual phosphorylation of the MAP kinases ERK1/2 [15-18]. We have recently shown that each of the three conserved tyrosine residues in murine LEPRb exhibits different signalling capacities in the insulinoma cell line HIT-T15 [18]. Tyr985 mediated leptin-induced activation of extracellular signal-regulated kinases 1 and 2 (ERK1/2), Tyr1077 induced tyrosyl phosphorylation of STAT5, and Tyr1138 was capable of activating STAT1, STAT3, and STAT5. Although STAT factors are well known to regulate transcription, the range of transcriptional targets responsive to leptin stimulation of pancreatic β-cells is not well characterised [19].

In the present study we used rat RINm5F insulinoma cells as a model system to study the changes in gene expression that leptin elicits in β-cells and to characterise the signalling pathway(s) by which the transciptional effects of leptin are mediated. In particular, we show that several inflammation-related genes were upregulated by leptin. Notably, some of these mRNAs were also induced by IL-1β, a pro-inflammatory cytokine thought to play a major role in β-cell destruction in type 1 diabetes mellitus. These data provide the first characterisation of leptin's effects on gene expression in a non-neuronal cell type.

## Results

### Identification of leptin-induced genes in RINm5F cells

We have previously employed RINm5F cells ectopically expressing LEPRb to characterise leptin signal transduction in an insulin-producing cell line [18]. In order to identify genes regulated by leptin in pancreatic β-cells, we compared gene expression in leptin-stimulated and non-stimulated RINm5F cells by differential hybridisation of high-density filter arrays containing 27,648 non-redundant rat cDNAs. From 33 transcripts that showed a strong differential signal after 16 h of leptin treatment, we selected 13 for further expression analysis by Northern blot hybridisation. Table 1 lists 8 transcripts that were confirmed to be upregulated by leptin. Five transcripts were identified as false positives (see Additional file 1 for the complete list).

In this study we focus on those genes potentially related to inflammatory processes. Four transcripts encode secreted acute phase proteins: fibrinogen-β, tissue-type plasminogen activator (tPA), pancreatitis-associated protein (PAP1), and lipocalin-2 [20]. Preprotachykinin-1 is the precursor of substance P, a mediator of neurogenic inflammation [21]. In addition, the mitochondrial enzyme manganese-dependent superoxide dismutase (MnSOD) has also been classified as an "acute phase protein" because it is upregulated in many inflammatory conditions [22].

### Costimulatory effects of IL-1β and leptin

**Figure 1:**
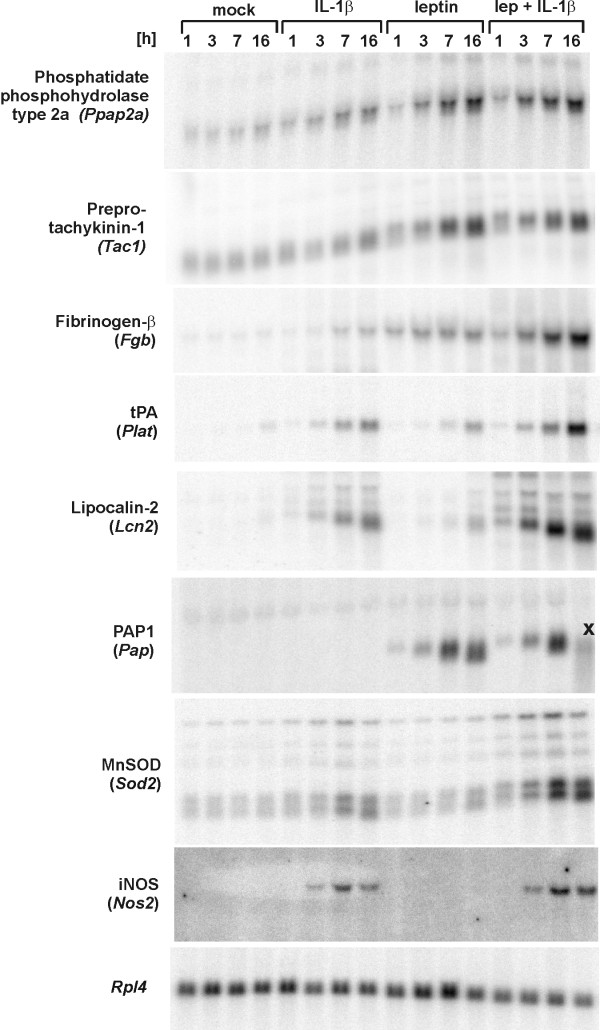
**Effects of leptin and IL-1β on inflammation-related transcripts in RINm5F insulinoma cells**. RINm5F cells stably expressing LEPRb were treated with leptin (100 ng/ml), IL-1β (50 u/ml), or a combination of both for the times indicated (1, 3, 7 or 16 h). Total RNA was isolated and subjected to Northern blot analysis with cDNA probes identified by differential hybridisation of the cDNA array (upper 6 panels, see table 1). iNOS and RPL4 serve as controls for an IL-1β-regulated mRNA and an unregulated mRNA, respectively. Gene symbols are shown in *italics*. A sample of degraded RNA on one of the blots is marked with an ***x***.

Considering that interleukin-1β (IL-1β) is an important inflammatory mediator in pancreatic β-cells, we asked whether leptin and IL-1β had overlapping effects on the expression of inflammation-related genes. Therefore, RINm5F cells were stimulated with IL-1β, leptin, or a combination of leptin and IL-1β for different time periods, and mRNA levels of specific transcripts were analysed by Northern blotting (Figure 1). As controls, we used probes for iNOS, which is known to be induced by IL-1β, and ribosomal protein L4 (RPL4) as a loading control.

According to the different time courses of leptin-induced upregulation, Northern blot analysis allowed us to distinguish immediate early (fibrinogen-β), delayed early (PAP1) and late target genes (lipocalin-2, preprotachykinin, tPA, MnSOD, and phosphatidate phosphohydrolase 2a/PPAP2A), which had their expression maxima at 1–3 h, 7 h and 16 h of leptin stimulation, respectively. Concerning the effect of IL-1β, mRNA levels for fibrinogen-β, tPA, lipocalin2, MnSOD and PPAP2A were found to be upregulated by IL-1β alone. Among them, lipocalin2 and MnSOD were more strongly induced by IL1β than by leptin. As expected, iNOS mRNA was induced by IL-1β but not by leptin. Costimulation with IL1β and leptin revealed synergistic effects of both cytokines on the expression of fibrinogen-β, lipocalin-2 and MnSOD, *i.e*. the effect of costimulation exceeded the added effects of each cytokine.

### Comparison of leptin and IFNγ

**Figure 2:**
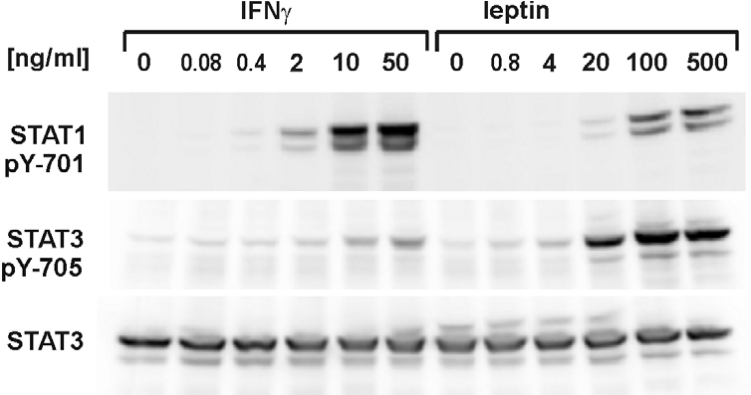
**Activation of STAT1 and STAT3 by leptin and IFNγ**. RINm5F cells stably expressing LEPRb were stimulated with the indicated concentrations of IFNγ or leptin for 15 min. Tyrosyl phosphorylation of STAT1 and STAT3 was determined by Western blot analysis of total cellular lysates with activation state-specific antibodies. The two bands reacting with the pTyr(705)-STAT1 antibody represent the splice variants STAT1α (91kD) and STAT1β (84 kD). The lower band detected by the STAT3 antibodies is probably the splice variant STAT3β. Total STAT3 immunoreactivity is shown as a control.

It is well known that IL-1β and IFNγ synergistically regulate inflammation-related genes in pancreatic β-cells [23]. Since both leptin and IFNγ modulate gene expression by activating the JAK/STAT pathway, we compared the effects of these cytokines on the activation of STAT factors in the RINm5F cell line. As we have previously reported [16], leptin induced tyrosyl phosphorylation of both STAT3 and STAT1 (Figure 2). In contrast, IFNγ rather selectively activated STAT1. Although in this direct comparison IFNγ elicited a stronger tyrosyl phosphorylation of STAT1, this result hints to the possibility that both cytokines have partially overlapping effects on gene expression in RINm5F cells.

**Figure 3:**
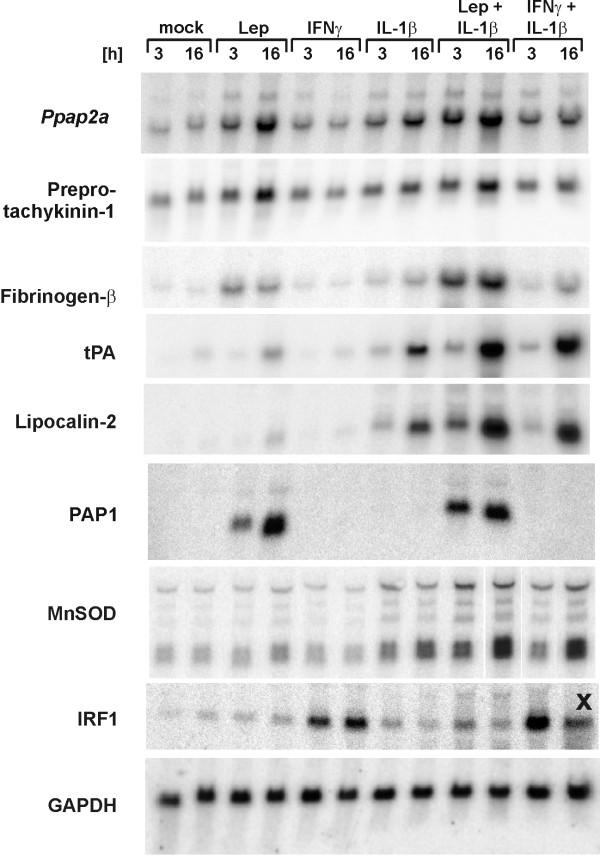
**Effects of leptin and IFNγ on inflammation-related transcripts**. RINm5F cells were treated with the indicated cytokines (100 ng/ml leptin; 50 u/ml IL-1β; 25 ng/ml IFNγ) or combinations thereof for 3 h or 16 h before total RNA was isolated. mRNA levels of the indicated genes were assessed by Northern blot hybridisation. GAPDH was used as a control. The RNAs from leptin and/or IL-1 β treated cells were obtained in an experiment independent from that shown in Figure 1. A sample of partially degraded RNA on one of the blots is marked with an ***x***.

Next we analysed the effects of leptin and IFNγ on the expression of the inflammation-related transcripts identified in the present study. RINm5F cells were stimulated with leptin or IFNγ either alone or in combination with IL-1β, and transcript levels were determined by Northern blot hybridisation. A probe for IFNγ-regulated factor 1 (IRF1) was included as a positive control for the action of IFNγ. As shown in Figure 3, IFNγ alone did not upregulate the transcripts that were induced by leptin. On the other hand, IRF1 mRNA levels were not affected by leptin. These results show that leptin and IFNγ differentially regulate the expression of inflammation-related genes in RINm5F insulinoma cells, probably because the cytokines act *via *different STAT factors. Interestingly, similar to leptin, IFNγ enhanced the IL-1β-induced upregulation of the mRNAs for tPA, lipocalin-2 and MnSOD.

### Requirement of Tyr-1138 in LEPRb for induction of inflammation-related genes

**Figure 4:**
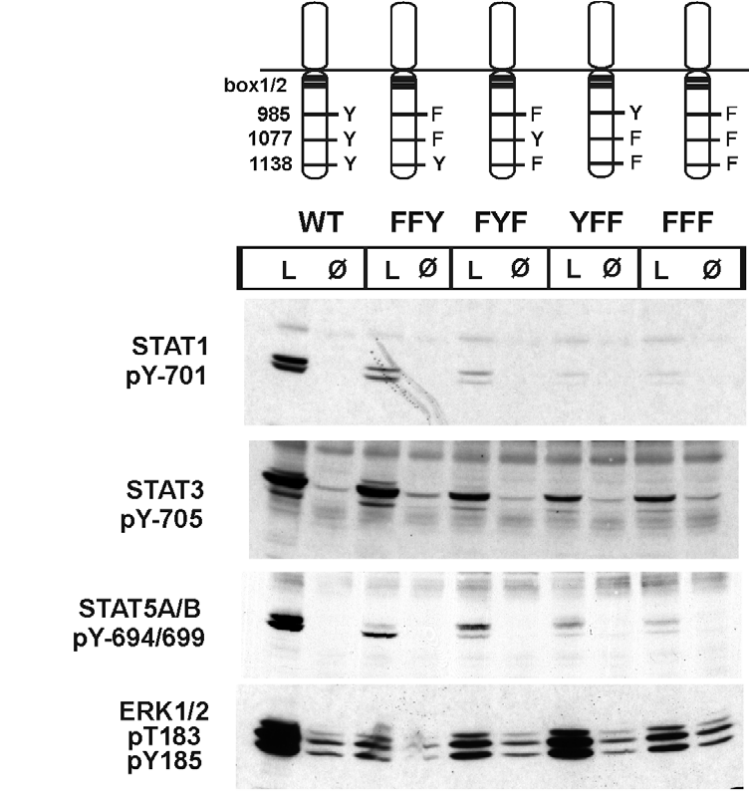
**Signalling capacity of RINm5F cells expressing LEPRb point mutants**. RINm5F cells stably expressing the wild type LEPRb (YYY) or the indicated LEPRb tyrosine/phenylalanine mutants were treated with 100 ng/ml leptin (L) or vehicle (∅) for 15 min before total cell lysates were prepared. Leptin-induced phosphorylation of downstream signalling molecules was assayed by Western blotting and immunodetection with phospho-specific antibodies.

Next, we asked which signalling pathway might be involved in the induction of the inflammation-related genes by leptin. To determine the role of the three intracellular tyrosine residues (Tyr985, Tyr1077, Tyr1138) in LEPRb-mediated activation of gene expression, we generated RINm5F cell lines that stably express LEPRb point mutants in which phenylalanines replace two of the three tyrosines, or the triple mutant. Leptin binding assays verified that all LEPRb constructs were expressed at the cell surface and differed by less than 2.2-fold in leptin binding (surface binding sites of mutant receptors relative to the wild type receptor: FFY, 1.10 ± 0.05; FYF 1.4 ± 0.06; YFF, 2.01 ± 0.13; FFF, 2.13 ± 0.47; for designations of the LEPRb mutants see Figure 4).

To characterise the signalling potential of these cells, we analyzed the leptin-induced phosphorylation of STAT factors and of ERK1/2 by Western blotting with activation state-specific antibodies (Figure 4). The results show that the full activation of STAT1 and STAT3 required the presence of Tyr1138, whereas the phosphorylation of STAT5 was mediated equally well by Tyr1077 and Tyr1138. The major part of ERK phosphorylation was dependent on the membrane-proximal tyrosine residue (Tyr985). These findings are in agreement with results that we have previously obtained in transiently transfected HIT-T15 insulinoma cells [18]. Intriguingly, a residual phosphorylation of STAT factors and of ERK1/2 was detectable even when all intracellular LEPRb tyrosine residues had been exchanged for phenylalanine. This cannot be explained by the action of endogenous LEPRb since neither the parental RINm5F cells nor control cells that were infected with the empty vector showed detectable effects of leptin (data not shown).

**Figure 5:**
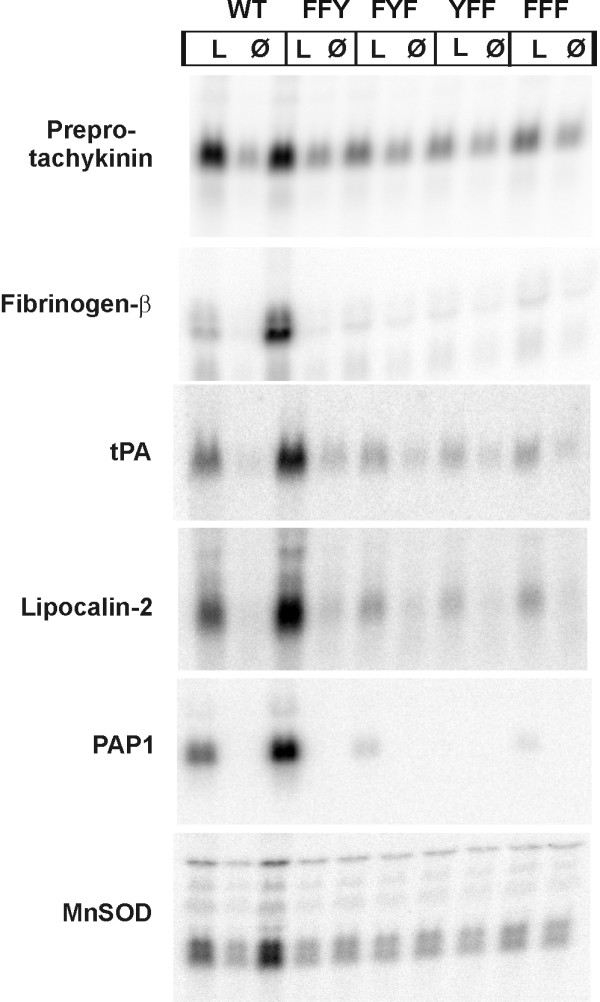
**Regulation of leptin target gene expression by LEPRb tyrosine mutants**. RINm5F cells expressing the indicated LEPRb tyrosine mutants were treated with 100 ng/ml leptin (L) or vehicle (∅) for 16 h. Northern blots were hybridised with the indicated cDNA probes.

These cell lines were now used to study the capacity of the LEPRb point mutants to upregulate mRNA levels of the leptin target genes identified in this work. As shown in Figure 5, induction by leptin depended on the presence of Tyr1138, suggesting that the transcription of these genes is regulated by STAT3 and/or STAT1. Consistent with the tyrosine-independent activation of STAT3 detected by Western blotting (Figure 4), weak leptin effects were also observed in the cell lines expressing LEPRb-YFF or LEPRb-FYF. However, the degree of induction by these constructs did not exceed that of the triple mutant (FFF). Interestingly, transcript levels were always higher in cells expressing LEPRb-FFY compared to wild type LEPRb, consistent with a role for Tyr985 and Tyr1077 in the negative regulation of leptin signalling [24,28].

### Leptin controls preprotachykinin-1 and lipocalin-2 gene expression at the level of promoter regulation

**Figure 6:**
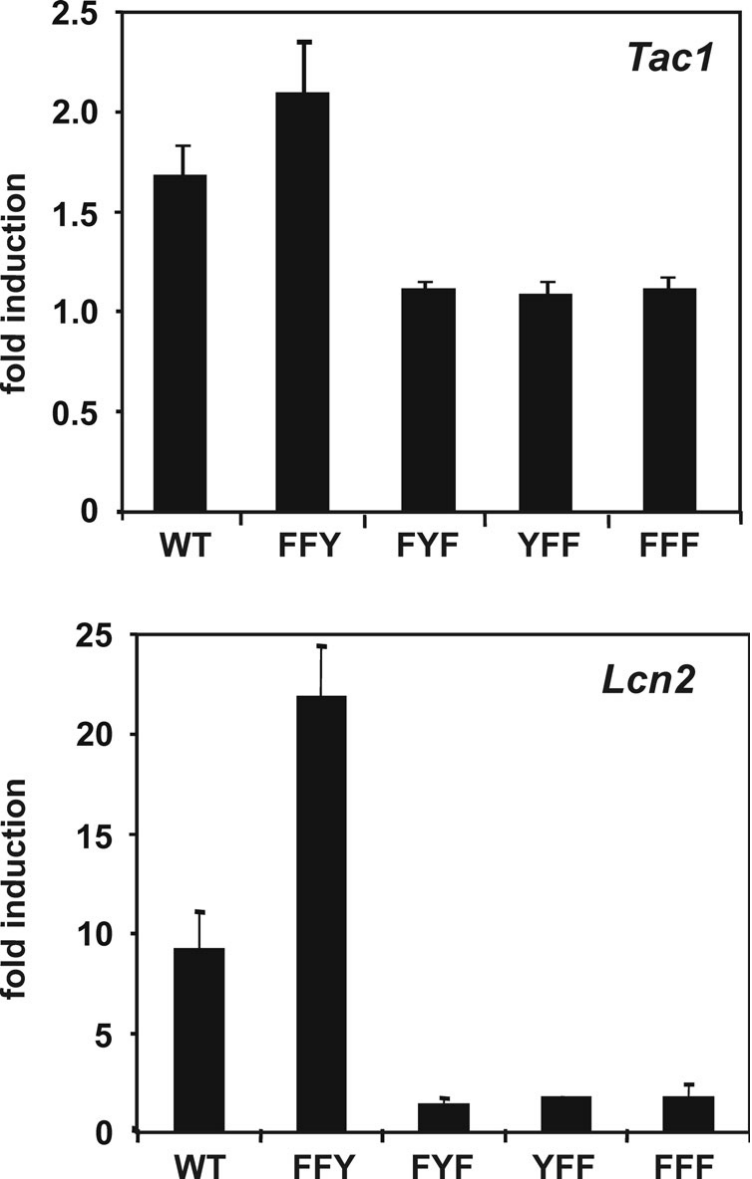
**Regulation of *Lcn2 *and *Tac1 *promoter by LEPRb mutants**. HIT-T15 hamster insulinoma cells were transiently transfected with luciferase constructs driven by the rat *Tac1 *promoter or the rat *Lcn2 *promoter, along with the indicated LEPRb expression plasmids. Twenty-four hours after transfection, the cells were treated with 100 ng/ml leptin or vehicle for 24 hours in serum-free medium. Luciferase activities were determined from duplicate wells and data were normalised to β-galactosidase activities. Data are expressed as fold stimulation by leptin relative to unstimulated cells. Bars reflect means +/- SD of three independent experiments (except for FFY in the upper panel which is the mean of two).

To substantiate the hypothesis that leptin controls the expression of inflammation-related genes by way of transcriptional regulation, we decided to determine the effect of leptin on promoter activity. We selected two rat genes whose promoters had not previously been analysed for regulation by STAT factors, *Lcn2 *(encoding lipocalin-2) and *Tac1 *(preprotachykinin-1). As in our previous studies [18], we used HIT-T15 hamster insulinoma cells for the promoter assays because RINm5F cells were only poorly transfected in transient assays. The results of the luciferase assays are presented in Figure 6 and show clearly that both the *Lcn2 *and the *Tac1 *promoter are regulated by leptin. Consistent with the Northern blot results, promoter induction depended on the presence of Tyr1138 in LEPRb, supporting the hypothesis that STAT1 and/or STAT3 are involved. Also in agreement with the Northern blot data, *Lcn2 *promoter activity was very low in the absence of leptin and was strongly induced by leptin, whereas the *Tac1 *promoter showed considerable basal activity in the absence of a stimulus. These data indicate that leptin upregulates expression of lipocalin-2 and preprotachykinin-1 by control of transcription.

**Figure 7:**
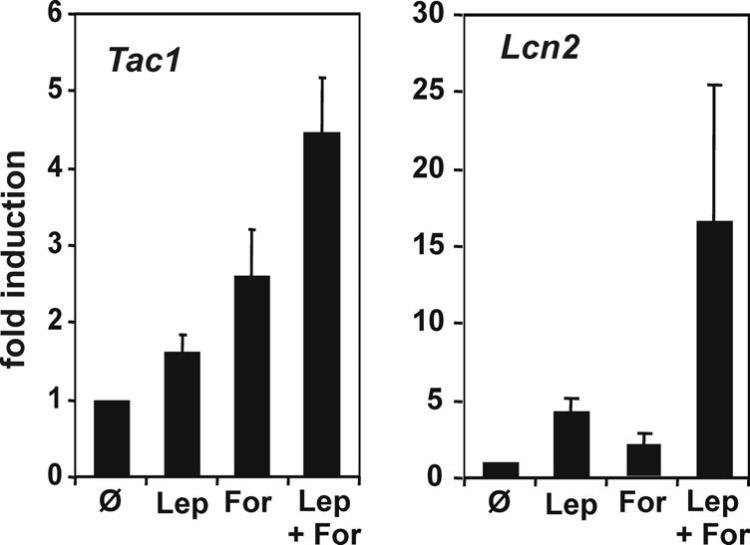
**Synergistic regulation of *Lcn2 *and *Tac1 *promoter activities by leptin and forskolin in PC-12 pheochromocytoma cells**. PC-12 cells stably expressing LEPRb were transiently transfected with the *Tac1 *or *Lcn2 *reporter gene constructs. Cells were left untreated or treated with leptin (100 ng/ml) and/or forskolin (10 μg/ml) for 24 hours before lysis. Luciferase activities were determined from duplicate wells and data were normalised to β-galactosidase activities. Data are expressed as fold stimulation by the treatment relative to unstimulated cells. Results are means +/- SD of three (*Tac1 *promoter) or four (*Lcn2*-promoter) independent experiments.

To address the question whether the regulation of these promoters by leptin was a specific feature of insulinoma cells, we took advantage of a LEPRb-expressing PC-12 pheochromocytoma cell line that was established in a previous study [25]. In this cell line, the effect of leptin on target gene promoters was found to be enhanced by simultaneous treatment with forskolin. Forskolin is a nonspecific activator of adenylate cyclases and enhances intracellular levels of cAMP. Similar to the results obtained in HIT-T15 cells transfected with wild type LEPRb, leptin induced both the *Tac1 *and the *Lcn2 *promoter in the PC-12 cell line (Figure 7). The weaker effect of leptin as compared to the HIT-T15 cells is likely due to the fact that the PC-12 cells were not kept serum-free during treatment with leptin and/or forskolin, in order to avoid differentiation of the cells. Treatment with forskolin further enhanced the effect of leptin on both promoters, as previously observed for other leptin-regulated promoters in this cell line (proopiomelanocortin, neuropeptide Y and PAP [26]).

### Leptin increases caspase-like proteolytic activity in RINm5F cells

**Figure 8:**
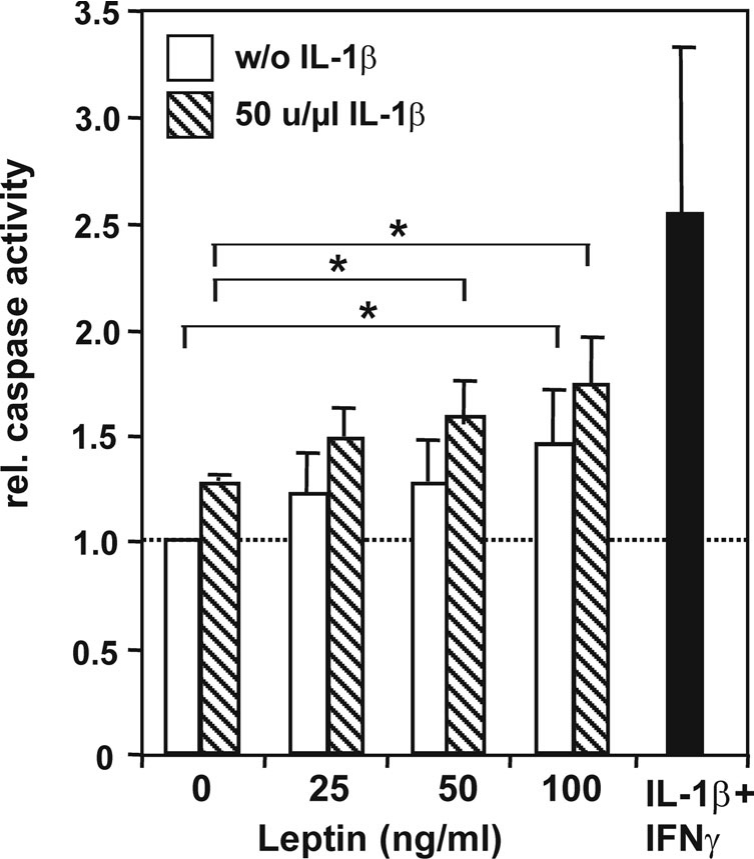
**Leptin induces caspase activity in RINm5F cells**. LEPRb-expressing RINm5F cells were exposed to the indicated concentrations of leptin for 48 h, alone (open bars) or in combination with 50 u/ml IL-1β (hatched). Alternatively, cells were treated with a mix of 50 u/ml IL-1β and 50 ng/ml IFNγ. Caspase activity was determined in total cell lysates as the sequence-specific cleavage of a proluminescent DEVD-aminoluciferin substrate. Data are expressed as fold stimulation by the cytokine treatment relative to unstimulated cells. Bars reflect means +/- SD of 4 independent experiments. * P < 0.05, treated *vs*. untreated. The effect of 50 u/ml IL-1β and 50 ng/ml IFNγ was not significantly greater than that of 50 u/ml IL-1β plus 100 ng/ml leptin (*P *= 0.058)

To assess a possible cytotoxic effect of leptin on insulin-producing cells, we determined the effect of leptin treatment on the enzymatic activity of caspase 3 (and caspase 3-like proteases) as a key player in apoptosis induction. Exposure of RINm5F-LEPRb cells to leptin led to caspase 3 activation in a dose-dependent manner (Figure 8). Co-administration of leptin with IL-1β revealed an additive effect of the cytokines. Leptin did not alter caspase activity in untransfected RINm5F cells (data not shown), indicating that the effect depended on the presence of LEPRb. As a positive control, the cells were also exposed to a mix of IL-1β and IFNγ which is known to be proapoptotic for RINm5F cells [27]. In this direct comparison, the combination of IL-1β (50 u/ml) and IFNγ (50 ng/ml) resulted in a stronger induction of caspase activity than IL-1β (50 u/ml) plus leptin (100 ng/ml).

## Discussion

To gain insight into the gene regulatory effects of leptin that may contribute to its action on body weight regulation, screens for the identification of leptin-regulated genes have previously been performed in neuronal cell lines overexpressing LEPRb [28,29]. Here we have used the insulin-producing cell line RINm5F as a model system to characterise the transcriptional effects of leptin in pancreatic β-cells, one of the peripheral target cells of leptin. A striking result of this screen is that leptin induces the expression of several genes related to inflammatory conditions, including genes encoding acute phase response proteins (tPA, fibrinogen-β, lipocalin-2, PAP1), preprotachykinin and MnSOD. Moreover, some of these leptin-induced transcripts were also induced by IL-1β, which is considered to be the most cytotoxic cytokine for β-cells [30,31]. These results reflect the molecular nature of leptin as a cytokine and are consistent with the hypothesis that high levels of leptin may play a role in the low-grade inflammatory state associated with obesity [32].

What might be the role of these inflammation-related proteins in pancreatic islets? Of the genes listed in table 1, only *Sod2 *(encoding MnSOD) has been functionally studied in insulin-producing cells. *Sod2 *is strongly induced in many cells, including RINm5F, by IL-1β *via *binding of NFκB to specific promoter elements [33]. Oxygen free radicals are generated in cytokine-stimulated β-cells, and overexpression of MnSOD, which inactivates mitochondrially derived oxygen free radicals, can prevent cytokine-induced apoptosis of β-cells [34-36]. Thus, upregulation of MnSOD by leptin is likely to have a protective effect on β-cells.

The role of the secreted acute phase proteins in β-cell inflammation has not yet been directly addressed. The product of the *Pap *gene (also known as PAP1 or Reg-2 in rat or Reg3β in mouse) has been designated "pancreatitis-associated protein" (PAP) because of its increased expression in rat pancreatic acinar cells in acute pancreatitis [37]. PAP1 belongs to a family of small secretory proteins that are related to REG1. The gene name *Reg1 *refers to regenerating pancreatic islets following partial pancreatectomy, where transcripts of *Reg1 *are significantly induced [38]. The REG1 protein is thought to act on pancreatic β-cells as an autocrine and/or paracrine factor and has been demonstrated to induce proliferation of β-cells and thereby to ameliorate the diabetes of 90% depancreatised rats and mice [39]. It is not known whether other members of the REG family share this ability, but PAP1 has been shown to act as an auto/paracrine neurotrophic factor on motoneurons [40]. Furthermore, PAP1 has anti-inflammatory effects in pancreatitis and inflammatory bowel disease [41], suggesting that it may also protect β-cells in islet inflammation. Interestingly, the *PAP *gene was found to be overexpressed in islets from a patient with recent-onset type 1 diabetes [42], indicating that its upregulation in islet inflammation is not restricted to rodents.

Lipocalin-2 (also known as 24p3, neutrophil gelatinase-associated lipocalin NGAL, siderocalin, uterocalin, superinducible protein 24) has been identified as a very highly regulated transcript in many inflammatory conditions [43]. Lipocalin-2 sequesters bacterial siderophores and is a bacteriostatic agent contributing to the innate immune response [44]. Furthermore, the iron-free lipocalin-2 apoprotein can induce apoptosis of several cell types, including leukocytes, whereas iron-containing holo lipocalin-2 can prevent apoptosis [45,46]. The effect of lipocalin-2 on pancreatic β-cells has not yet been studied.

Fibrinogen-β and tPA are classical acute phase plasma proteins that are predominately expressed in the liver [20]. It is unlikely that the enhanced expression of these proteins in pancreatic islets contributes significantly to their blood level, but local effects on fibrogenesis or fibrolysis may occur in insulitis. Similarly, the physiological significance of preprotachykinin expression is not clear. Preprotachykinin-1 is the precursor of substance P, a mediator of neurogenic inflammation in inflammatory diseases of the respiratory, gastrointestinal, and musculoskeletal systems [21]. RINm5F cells have previously been described to express *Tac1 *and to secrete substance P [47]. Interestingly, substance P has recently been implicated in the autoimmune diabetes of the NOD mouse model, because expression of the receptor for substance P (NK-1 receptor) was upregulated in the islets during development of insulitis [48]. However, preprotachykinin has been reported to be expressed in rat pancreatic islets only transiently during development, and the expression in RINm5F cells may reflect embryonal characteristics of this cell line [49].

In this study, we took advantage of the RINm5F insulinoma cell line as a model for pancreatic β-cells because cultured clonal cells provide a homogenous, well defined experimental system for array experiments. RINm5F cells have frequently been used to characterise the effect of cytokines on insulin-producing cells (*e.g*. [36,50]). We have recently shown that RINm5F cells expressing the long splicing variant of the leptin receptor (LEPRb) display the full range of leptin effects relevant for transcriptional regulation (activation of STAT1, STAT3, STAT5, ERK1/2; [18]. Importantly, most of the leptin-induced genes detected in the present screen have previously been identified in microarray analyses of animal models of type 1 or type 2 diabetes or in isolated rat pancreatic β-cells. *Pap *and *Lcn2 *were identified as the genes most strongly upregulated in islets from diabetic GK rats (a model for type 2 diabetes) [51]. Expression of *Pap*, *Lcn2*, and *Sod2 *(encoding MnSOD) was found to be increased in a mouse model of autoimmune diabetes [52]. Transcripts for tPA, fibrinogen-β and MnSOD were induced by IL-1β in isolated rat βcells [23]. Taken together, these results show that in RINm5F cells leptin induces a set of genes that is also expressed in β-cells in models of diabetes and β-cell destruction.

To sum up, the set of leptin-induced transcripts characterised here includes both mRNAs for protective (MnSOD, PAP1) as well as potentially cytotoxic proteins (lipocalin-2, preprotachykinin). Other well-characterised proinflammatory cytokines, such as the combination of IL-1β and IFNγ, are also known to induce defence-related genes [23]. Leptin did not induce the same genes as IFNγ, but both cytokines had the same costimulatory effect on certain IL-1β-induced transcripts (tPA, lipocalin-2, MnSOD; see Figure 3). After all, leptin induced caspase activation in RINm5F cells, strongly suggestive of a pro-apoptotic effect of leptin. Notably, the leptin concentration applied (100 ng/ml) is within the range of blood levels observed in obese humans [53]. However, more work is needed to establish whether the high levels of leptin in obesity have a protective effect or contribute to islet destruction *in vivo*.

Finally, we have employed LEPRb tyrosine/phenylalanine point mutants to provide an initial characterisation of the signalling pathway responsible for leptin's effect on gene expression in insulin-producing cells. For all genes under investigation, the induction by leptin was dependent on the presence of Tyr1138 which is embedded in a canonical box3 motif (Tyr-X-X-Gln). This result suggests that the effect of leptin requires the activation of STAT3 and/or STAT1. Some of the genes have previously been shown to be regulated by cytokines that activate STAT3, including MnSOD, fibrinogen-β, tPA and PAP1. We have previously identified *Pap *as a leptin-induced gene in rat PC12 pheochromocytoma cells, and have defined a STAT3-responsive promoter element [25,26]. *Lcn2 *is also known to be induced by cytokines that transmit their signal *via *activation of the JAK/STAT pathway (IL-9, IL-17, G-CSF). Our data show that induction of *Lcn2 *by leptin depends on the presence of the Tyr1138 in LEPRb, suggesting that activation of STAT3 and/or STAT1 is required. However, the rat *Lcn2 *promoter contains no consensus STAT-binding element within the sequence tested in the reporter gene assay (search of the TRANSFAC database using the TESS software [54]). Similarly, no recognisable STAT3-binding site is present in the rat *Tac1 *promoter. Considering the delayed response of both the lipocalin-2 and preprotachykinin-1 mRNA to leptin, it appears likely that regulation of these genes occurs by an indirect mechanism.

## Conclusion

The main result of our study is that leptin induces the expression of inflammation-related genes in RINm5F insulinoma cells, of which only PAP was already known as a leptin target gene. Several of these genes have also been described to be upregulated in animal models of diabetes (see discussion section), suggesting that this pattern of changes in gene expression reflects an inflammatory response of β-cells. The transcriptional effects of leptin required a functional box3 motif of the leptin receptor and are likely mediated *via *STAT3. Intriguingly, the effect of leptin on gene expression overlaps partially with that of IL-1β, a cytokine acting via a completely different signalling pathway, but not with that of IFNγ, which activates STAT1. The observation that leptin synergises with IL-1β in caspase 3 induction is consistent with the proposed function of leptin as a modulator of immune and inflammatory responses.

## Methods

### Cell culture, transient transfections and stably transfected cell lines

Rat RINm5F insulinoma cells were grown in RPMI 1640 medium with L-glutamine, 10% (v/v) fetal bovine serum, 100 units/ml penicillin, and 100 mg/ml streptomycin at 37°C and 5% CO_2_. HIT-T15 hamster insulinoma cells were cultivated under the same conditions except that 5% horse serum was added to the medium. Rat PC-12 pheochromocytoma cells stably expressing wild type LEPRb [25] were grown in DMEM supplemented with 5% fetal calf serum and 10% horse serum on collagen-coated culture dishes. To obtain single cells for plating, cells were passed through a syringe with a 22-gauge needle. HIT-T15 and PC-12 cells were transiently transfected by the polyethylenimine method (Jet-PEI reagent; Polyplus-Transfection, Illkirch, France). The RINm5F cell line stably expressing wild type LEPRb has been described previously [18]. For stable expression of LEPRb tyrosine/phenylalanine mutants, the mutant cDNAs were subcloned from the pMET7 vector constructs [28] into the retroviral vector pWZLNeo. These mutant constructs and the corresponding wild type receptor used in the experiments shown in Figures 4, 5, 6 contain a C-terminal myc-tag. Confluent RINm5F cells were infected with pWZL-Neo viruses, and infected cells were selected with geneticine sulphate (G418; 1 mg/ml) as described before [18]. Cell surface expression of the different LEPRb constructs on RINm5F cells was measured using a mouse leptin-secreted alkaline phosphatase chimeric protein as described [28]. Murine leptin was purchased from PeproTech (London, UK), human IL-1β from Roche Diagnostics (Mannheim, Germany), and rat IFNγ from Serotec (Düsseldorf, Germany).

### Isolation of RNA and differential hybridisation of high density cDNA filter arrays

RINm5F-LepRb cells grown to 80% confluence were incubated in serum-free medium for 20 h before they were stimulated with murine leptin (100 ng/ml; PeproTech, London, UK) or left unstimulated for 16 h. Total RNA was isolated using the RNeasy Midi Kit (Qiagen, Hilden, Germany), and poly-A^+^-RNA was prepared using oligo-(dT)_30_-coupled paramagnetic beads (Chemagen, Baesweiler, Germany). Complex cDNA probe was generated from 500 ng of poly-A^+^-RNA by reverse transcription in the presence of 7.5 MBq [α-^33^P]-dCTP (111 TBq/mmol Hartmann Analytics, Braunschweig, Germany) according to the protocol suggested by the manufacturer of the cDNA array (RZPD Berlin, Germany). The ^33^P-labelled single-stranded cDNA was hybridised to high density filter arrays that contained double-spotted, PCR-amplified cDNA inserts of the rat UniGene cDNA library (library 953, RZPD Berlin). This library comprises a set of 27,648 non-redundant rat cDNA clones provided by Bento Soares (University of Iowa). The filter were washed at high stringency (final wash in 150 mM NaCl/15 mM Na-citrate pH 7.0/0.1% SDS at 65°C) and exposed for 72 hours to a phosphor storage screen (Fujifilm Imaging Plate BAS-MS2325, Fuji Photo Film, Japan). For probe removal, the filter was doused with boiling 0.1% SDS, 5 mM phosphate buffer (ph 7.6) and left at room temperature under continuous agitation. This procedure was repeated and probe removal confirmed by exposure of an phosphor storage screen before rehybridization.

### Analysis of array data

Signal intensities were evaluated using the AIDA™ software (AIDA Advanced Image Data Analyzer and AIDA Array Compare V 3.0; Raytest, Straubenhardt, Germany) as follows: integrated intensity values were determined for each spot and means of the two spots containing the same cDNA probe were calculated. For background correction, the signal intensity of the space between the spots was subtracted. To normalise for global intensity differences between the data sets, intensity values were divided by the intensity value of the spots containing β-actin cDNA. mRNA levels of β-actin were confirmed by Northern blot analysis to be unaffected by leptin treatment of RINm5F cells (data not shown). Only transcripts with an absolute signal intensity ≥ 1.0 that showed a ≥ 2.5-fold increase were considered relevant. The data presented in Table 1 and and Additional file 1 were obtained by successive hybridisation of the same filter array with the two complex cDNA probes derived from one experiment. Confirmatory Northern blot hybridisations (last column of table in Additional file 1) were done with RNA samples from independent experiments. Potentially interesting transcripts were arbitrarily selected for further analysis.

### Northern blot analysis

Samples (10 μg) of total RNA were separated by denaturing formaldehyde electrophoresis on 1% (w/v) agarose gels and transferred by capillary blot onto positively charged nylon membranes (Hybond N+; Amersham, Freiburg, Germany). Labelled probes were generated from the inserts of cDNA clones listed in Table 1 by random oligonucleotide priming. Murine cDNA clones were used to generate the probes for iNOS (inducible nitric oxide synthase; IMAGE clone 3583251, GenBank acc. No. BE372884) and IRF-1 (interferon regulatory factor 1, IMAGE clone 3600525, GenBank acc. No. BC003821). Blots were re-hybridised with other cDNA probes after probe removal by incubation with 0.5% SDS at 100°C.

### Western blot analysis

Stably transfected RINm5F cells were incubated in serum-free medium for 18–22 h before cytokines (100 ng/ml leptin, 50 u/ml IL-1β, or 25 ng/ml IFNγ) were added for 15 min. Cells were washed with phosphate buffered saline and lysed in 1% SDS, 20 mM Tris-Cl pH 7.4 in a boiling water bath for 5 min. Total cellular lysates were separated by SDS-PAGE (8% gels), blotted on-to nitrocellulose, and phosphorylation of specific proteins was analysed using the following antibodies (Cell Signaling Technology, Beverly, MA): anti-p(Y701)-STAT1, anti-p(Y705)-STAT3, anti-p(Y694/699)-STAT5A/B, and anti-pT/pY-ERK1/2. Horseradish peroxidase-labelled anti-rabbit IgG (IgG-POD from Pierce Chemical Co., Rockford, IL) was used as the second antibody. Blots were re-used after stripping the primary antibody by incubation at room temperature for 20–30 min in 2% SDS, 50mM Tris-Cl, 150 mM NaCl, pH7.4 in the presence of 100 mM 2 mercaptoethanol.

### Reporter gene assays

A luciferase reporter construct containing the promoter region (-865 to +447; pSUB201-LPPT) of the rat preprotachykinin A gene (*Tac1*) was kindly provided by John Quinn, University of Liverpool, UK [55,56]. A 976-bp fragment of the rat lipocalin-2 (*Lcn2*) promoter region (-930 to +46, the first nucleotide of the cDNA sequence with GenBank acc. No. X13296 was defined as +1) was amplified with primers rLcn2-930for (5'-ATAGGTACCGGATGCCTTTTCAAGGGAGG-3') and rLcn2-Exon1rev (5'-GACACCCAGGCCCATGGTTTCAGC-3') from rat genomic DNA. The PCR product was cloned into the pGL3basic vector (Promega, Madison, WI) using the engineered *Kpn*I site in the upstream primer and an endogenous *Nco*I site at the initiator codon, resulting in pGL3-rLcn2(-930). DNA sequencing confirmed the identity of the PCR product and revealed a polymorphism in the rat *Lcn2 *promoter: a dinucleotide repeat (at -846 to -784) in our construct (from RINm5F genomic DNA) contained two more CA dinucleotides than the sequence (from the Sprague Dawley rat strain) available at the Genome Browser Gateway [57]. This polymorphic microsatellite has previously been used to map the rat *Lcn2 *gene [58]

HIT-T15 cells were seeded in six-well plates (3.3–4.0 × 10^5 ^cells per well) and transfected with 0.5 μg of pSUB201-LPPT or pGL3- rLcn2(-930), along with 1.0 μg of the β-galactosidase reporter control plasmid pSVβ-gal (Promega) and 0.3 μg of the pMET7-LEPRb expression plasmids [28]. 24 h or 48 h after transfection, the cells were stimulated with 100 ng/ml leptin for 22 hours in serum-free medium. Luciferase activities were determined from duplicate wells with the help of a commercial kit (Promega) and data were normalised to β-galactosidase activities. For assays of PC-12-L8 cells, cells from one 80 cm^2^-flask were seeded into 12 wells of collagen-coated six-well plates and transfected with reporter gene plasmids as above. Fresh serum-containing medium was added the day after transfection, and cells were treated with leptin (100 ng/ml) and/or forskolin (10 μM) 24 h later. To avoid effects of differentiation, care was taken that the cells never reached confluence.

### Assay of caspase activity

RINm5F cells were seeded in a white, non-translucent 96-well plate (10,000 cells per well). The following day, cells were supplied with fresh medium and stimulated with cytokines as indicated. After incubation for 72 h, DEVD-directed protease activity was determined from triplicate wells with the help of the Caspase-Glo™ 3/7 Assay (Promega) which detects caspase activity based on the sequence-specific cleavage of a proluminescent caspase-3/7 DEVD-aminoluciferin substrate. The data were evaluated for statistical significance with the two-tailed Student's *t *test, and the differences were considered significant at *P *< 0.05.

## Abbreviations

ERK, extracellular signal-regulated kinase; iNOS, inducible nitric oxide synthase; IL, interleukin; IRF1, interferon regulatory factor 1; LEPR, leptin receptor; MnSOD, manganese-dependent superoxide dismutase; PAP, pancreatitis-associated protein; STAT, signal transducer and activator of transcription; tPA, tissue-type plasminogen activator

## Authors' contributions

PH carried out most of the experiments and drafted the manuscript. JZ established and characterized the stable RINm5F cell lines. SK and HK performed the reporter gene assays in HIT-T15 cells and carried out some of the Northern blots. SBL carried out the caspase assays and experiments with the PC12 cells. LZ constructed the LEPR expression clones and performed the binding assay. JT established the PC12 cell system and participated in the interpretation of the results and final editing of the manuscript. WB conceived of and planned this study and edited the manuscript. All authors read and approved the final manuscript.

## Supplementary Material

Additional File 1 Complete list of potentially leptin-induced transcripts identified by differential hybridisation. Leptin-induced cDNAs were identified in 16-h leptin-treated RINm5F cells as described in the methods section. This table lists all non-redundant cDNAs that exhibit a signal intensity ≥ 1 and were induced by ≥ 2.5 fold. Click here for file

## Figures and Tables

**Table 1:** List of transcripts upregulated by 16 h-leptin treatment in RINm5F cells

				**Signal intensities**^†^		
					
	Gene symbol*	**GenBank Accession**.	**UniGene**	**mock**	**leptin**	**Fold induction by leptin**
Phosphatidate phosphohydrolase 2a	*Ppap2a*	AI555126	Rn.61687	0.3	2.4	8.9
Regulator of G-protein signaling 4	*Rgs4*	AI502324^‡^	Rn.11065	3.2	16.9	5.2^‡^
Preprotachykinin 1	*Tac1*	AA818532	Rn.1920	44.6	188.2	4.2
Fibrinogen, beta polypeptide	*Fgb*	AI059690^§^	Rn.11416	3.0	11.4	3.8^§^
Plasminogen activator, tissue-type (tPA)	*Plat*	AA875008	Rn.107102	2.3	7.7	3.3
Lipocalin 2	*Lcn2*	AI137617	Rn.11303	2.7	8.7	3.2
Pancreatitis-associated protein (PAP1)	*Pap*	AA998280	Rn.9727	1.9	5.5	2.9
superoxide dismutase 2 (MnSOD)	*Sod2*	AI029806	Rn.10488	17.1	48.3	2.8

* Official gene symbol in the Rat Genome Database

^† ^Phosphoimager evaluation of filter array hybridization (relative units corrected to background and normalized to β actin

^‡ ^Representative of four different Rgs4 cDNA clones (ratios: 5.2, 2.9, 2.8 and 2.4).

^§ ^Representative of two different fibrinogen-β cDNA clones (ratios: 3.8 and 2.8).
