# Supplementary material for: Leptin induces inflammation-related genes in RINm5F insulinoma cells
Source: BMC Mol Biol. 2007 May 23;8:41. doi: 10.1186/1471-2199-8-41 (PMC1890559; doi:10.1186/1471-2199-8-41)
Supplement: Additional File 1 — Complete list of potentially leptin-induced transcripts identified by differential hybridisation. Leptin-induced cDNAs were identified in 16-h leptin-treated RINm5F cells as described in the methods section. This table lists all non-redundant cDNAs that exhibit a signal intensity ≥ 1 and were induced by ≥ 2.5 fold. [file 1471-2199-8-41-S1.pdf]

Table A1 (additional material): Leptin-induced cDNAs were identified in 16-h leptin-treated RINm5F cells as described in the methods section. This table lists all non-redundant cDNAs that exhibit a signal intensity ≥ 1 and were induced by ≥ 2.5 fold.

GenBank Acc. No.	UniGene	Gene product (Gene symbol)	Signal intensity*		fold induction	Northern blot§
			MOCK	LEPTIN		
**AI555126**	**Rn.61687**	**Phosphatidate phosphohydrolase 2a (Ppap2a)**	**0.3**	**2.4**	**8.9**	**+**
**AI501010**	**Rn.1133**	**Ribosomal protein L4 (Rpl4)**	**1.4**	**9.4**	**6.9**	**-**
AI549418	Rn.1022	Coated vesicle membrane protein (Rnp24)	1.5	9.2	6.3	ø
**AI502324**	**Rn.11065**	**Regulator of G-protein signaling 4 (Rgs4)**	**3.2**	**16.9**	**5.2**	**+**
AI454367	Rn.35935	ADP ribosylation factor 4 (Arf4)	0.5	2.7	5.0	ø
AA859174	Rn.39743	RT1 class Ib gene (Aw2)	2.0	8.8	4.5	-
**AA818532**	**Rn.1920**	**Tachykinin 1 (Tac1)**	**44.6**	**188.2**	**4.2**	**+**
AI070457	Rn.965	Eukaryotic translation elongation factor 1 alpha 1 (Eef1a1)	2.9	11.9	4.2	ø
**AI059690**	**Rn.11416**	**Fibrinogen, beta polypeptide (Fgb)**	**3.0**	**11.4**	**3.8**	**+**
AI556900	Rn.106964	Similar to RIKEN cDNA C130022K22 gene	2.9	10.9	3.7	ø
AA964070	Rn.94848	Metastasis associated 3 (predicted)	1.2	4.4	3.7	ø
AI709970	Rn.102058	heat shock protein 60 liver (Hsp60)	2.0	7.1	3.6	-
AA859196	Rn.37575	tropomyosin isoform 6 (Tpm3)	4.1	14.0	3.4	ø
**AA875008**	**Rn.107102**	**plasminogen activator, tissue-type (Plat)**	**2.3**	**7.7**	**3.3**	**+**
AA964758	Rn.10426	Glycogen synthase kinase 3 beta (Gsk3b)	0.5	1.5	3.3	ø
AI059441	Rn.138883	Similar to adenylosuccinate synthase	0.6	1.9	3.2	ø
**AI137617**	**Rn.11303**	**Lipocalin 2 (Lcn2)**	**2.7**	**8.7**	**3.2**	**+**
AA965154	Rn.4225	14-3-3 epsilon (Ywhae)	7.2	21.1	2.9	ø
AI045008	Rn.2022	Ubxd2: UBX domain containing 2	2.4	7.0	2.9	ø
AI060102	Rn.28251	Rnf149: Ring finger protein 149	0.7	2.1	2.9	ø
AA957818	Rn.54447	ferritin, heavy polypeptide 1 (Fth1)	6.9	19.7	2.9	ø
**AA998280**	**Rn.9727**	**pancreatitis-associated protein (Pap)**	**1.9**	**5.5**	**2.9**	**+**
AA859496	Rn.28195	GTP cyclohydrolase 1 (Gch)	0.6	1.8	2.8	-
**AI029806**	**Rn.10488**	**superoxide dismutase 2, mitochondrial (Sod2)**	**17.1**	**48.3**	**2.8**	**+**
AI113089	Rn.102412	Destrin (Dstn)	5.5	15.6	2.8	ø
AA875006	Rn.32200	„transcribed locus"	2.1	5.7	2.7	ø
AA858597	Rn.4233	Similar to ARP2/3 complex 21 kDa subunit	1.2	3.2	2.7	ø
AA859695	/	No database entry yet	4.9	12.9	2.6	ø
AI029638	Rn.33275	Serine/threonine kinase 2 (Slk)	0.7	1.7	2.6	ø
AI454448	Rn.11527	Glucose regulated protein, 58 kDa (Grp58)	1.7	4.4	2.6	ø
AI043642	Rn.103382	Mal, T-cell differentiation protein 2 (Mal2)	1.2	3.0	2.6	ø
AA963208	Rn.2119	Ribosome associated membrane protein 4 (RAMP4)	2.7	6.8	2.6	-
AA858626	Rn.119131	cDNA clone IMAGE:7321089	8.1	20.9	2.6	ø

* signal intensities normalized to beta actin

§ Leptin induction of selected mRNAs was tested by Northern blot analysis of RNA from an independent experiment. Upregulation by leptin was confirmed (+), not confirmed (-), or not tested (ø).
